# Supplementary material for: Lung gene expression signatures suggest pathogenic links and molecular markers for pulmonary tuberculosis, adenocarcinoma and sarcoidosis
Source: Commun Biol. 2020 Oct 23;3:604. doi: 10.1038/s42003-020-01318-0 (PMC7584606; doi:10.1038/s42003-020-01318-0)
Supplement: Supplementary file 1 — Supplementary Information [file 42003_2020_1318_MOESM1_ESM.pdf]

Supplementary Materials

Lung gene expression signatures suggest pathogenic links and molecular markers for pulmonary tuberculosis, adenocarcinoma and sarcoidosis

Qiyao Chai[1,2,5], Zhe Lu[1,2,5], Zhidong Liu[3,5], Yanzhao Zhong[1,2], Fuzhen Zhang[1], Changgen Qiu[1,2], Bingxi Li[1], Jing Wang[1], Lingqiang Zhang[4], Yu Pang[3*] and Cui Hua Liu[1,2*]

[1]CAS Key Laboratory of Pathogenic Microbiology and Immunology, Institute of Microbiology, Center for Biosafety Mega-Science, Chinese Academy of Sciences, Beijing 100101, China. [2]Savaid Medical School, University of Chinese Academy of Sciences, Beijing 101408, China. [3]Beijing Tuberculosis and Thoracic Tumor Research Institute, Beijing Chest Hospital, Capital Medical University, Beijing 101149, China. [4]State Key Laboratory of Proteomics, Beijing Proteome Research Center, National Center of Protein Sciences (Beijing), Beijing Institute of Lifeomics, Beijing 100850, China. [5]These authors contributed equally: Qiyao Chai, Zhe Lu and Zhidong Liu.

[*]email: pangyupound@163.com; liucuihua@im.ac.cn

Supplementary Fig. 1

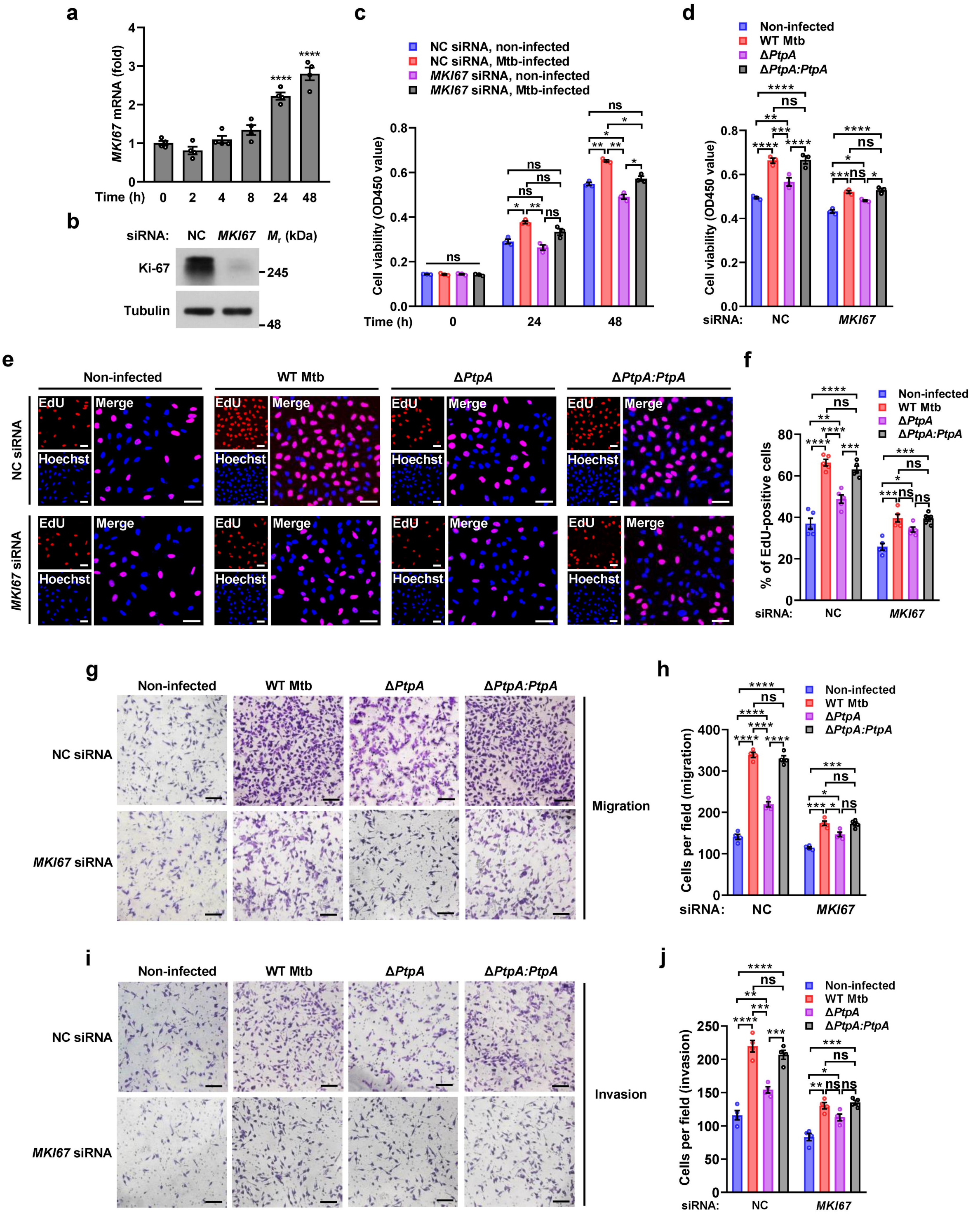

Supplementary Fig. 1 Mtb PtpA promotes tumor cell proliferation, migration and invasion partially depending on *MKI67*. a Quantitative PCR analysis of *MKI67* mRNA in A549 cells. Cells were infected with Mtb for 0–48 h. **b** Immunoblot analysis of Ki-67 and Tubulin from lysates of A549 cells transfected with negative control (NC) or *MKI67* siRNA for 24 h. **c**, **d** CCK-8 analysis of A549 cells. Cells were transfected with NC or *MKI67* siRNA as in **b**, and infected with or without wild-type Mtb for 0–48 h (**c**), or each of the indicated Mtb strains for 48 h (**d**). **e**, Representative images of EdU proliferation assay of A549 cells. Cells were transfected with NC or *MKI67* siRNA as in **b** and were infected with the indicated Mtb strains or not for 24 h. The proliferating cells were labeled with incorporated EdU-594 (red), and nuclei were stained with Hoechst 33342 (blue). Scale bars, 50 μm. **f** Quantification of EdU-positive cells as in **e**. Five independent visual fields were examined. **g**, **i** Transwell migration (**g**) and invasion (**i**) assays of A549 cells. Cells were transfected with NC or *MKI67* siRNA for 24 h and were infected with or without Mtb H37Rv, and were then allowed to migrate or invade for 12 h. The cells that had moved through the filter into the lower wells were stained with Crystal Violet. Scale bars, 100 μm. **h**, **j** Quantification of cells that had migrated (**h**) or invaded (**j**) through the filter. Four independent visual fields were examined. $P > 0.05$, not significant (ns); *$P < 0.05$; **$P < 0.01$; ***$P < 0.001$; ****$P < 0.0001$ (mean ± s.e.m. of $n = 4$ in **a**, **h**, and **j**, $n = 3$ in **c** and **d**, and $n =$ **5** in **f**, two-way ANOVA). All experiments were repeated at least three times independently.

Supplementary Fig. 2

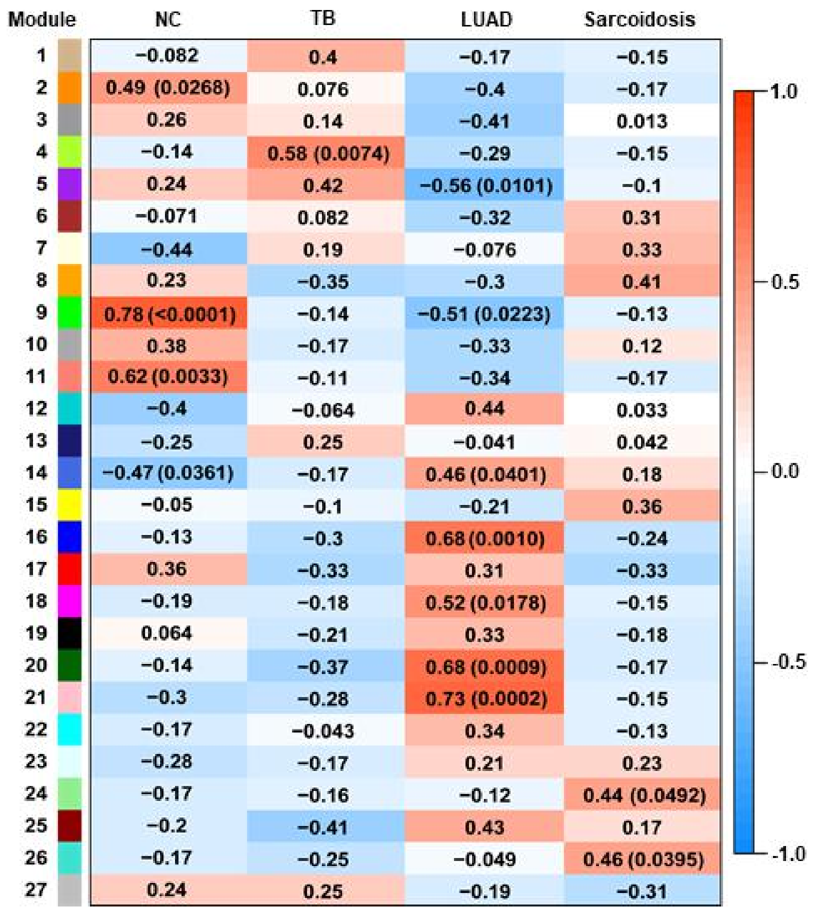

Supplementary Fig. 2 Weighted correlation network analysis identifies specific gene modules correlated to tuberculosis, lung adenocarcinoma or sarcoidosis. Each row in the table corresponds to a consensus gene module (1 to 27), and each column corresponds to a trait: normal control (NC), tuberculosis (TB), lung adenocarcinoma (LUAD) or sarcoidosis. Numbers in the table indicate the correlations of the corresponding module eigengenes and the studied traits, and *P*-values are shown in parentheses (only those *P*-values < 0.05 are shown). The table is color-coded based on correlation according to the color legend on the right. Module 4 (yellow-green), module 21 (pink) and module 26 (turquoise) are most significantly and positively correlated with TB, LUAD and sarcoidosis, respectively.

Supplementary Fig. 3

a

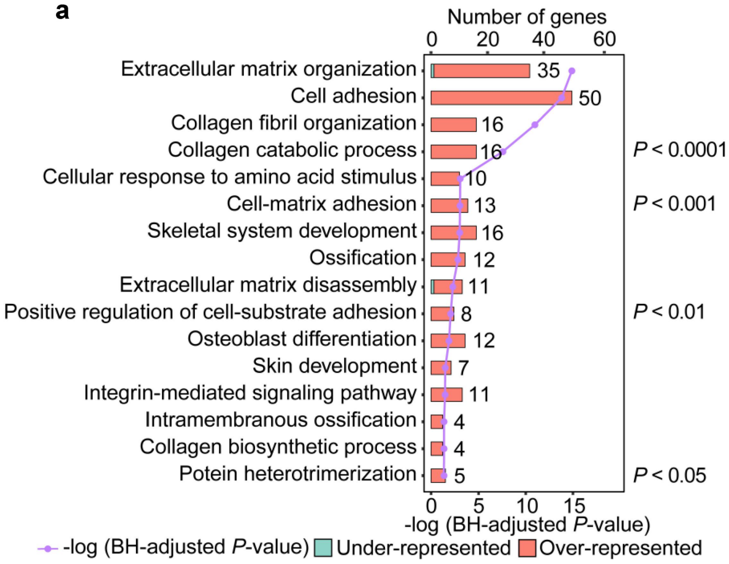

b

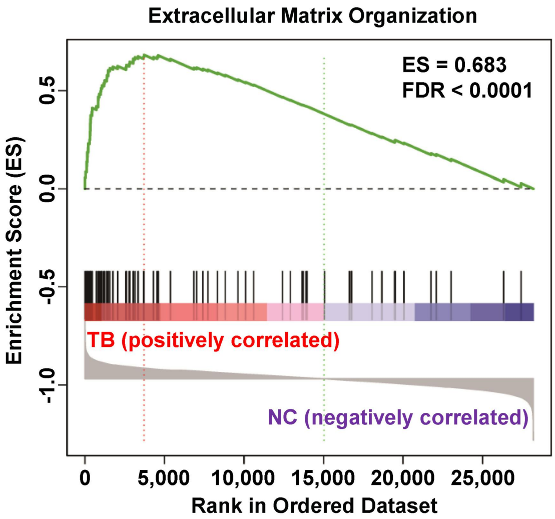

Supplementary Fig. 3 Enrichment of extracellular matrix (ECM) organization-related genes over-represented in TB lung tissues. a Gene Ontology (GO) analysis showing the enrichment of ECM organization-related genes in TB-correlated WGCNA gene module 4. The bars represent the abundance of under-represented (blue) and over-represent genes (red) in each biological process. The purple line represents Benjamini-Hochberg (BH)-adjusted *P*-value for each biological process. **b** Gene set enrichment analysis (GSEA) of TB and NC lung-tissue transcriptional profiles showing the positive correlation of TB patients with ECM organization-related gene signatures. The bar-code plot indicates the positions of genes in the gene set, and red and purple bars represent upregulated and downregulated genes, respectively.

Supplementary Fig. 4

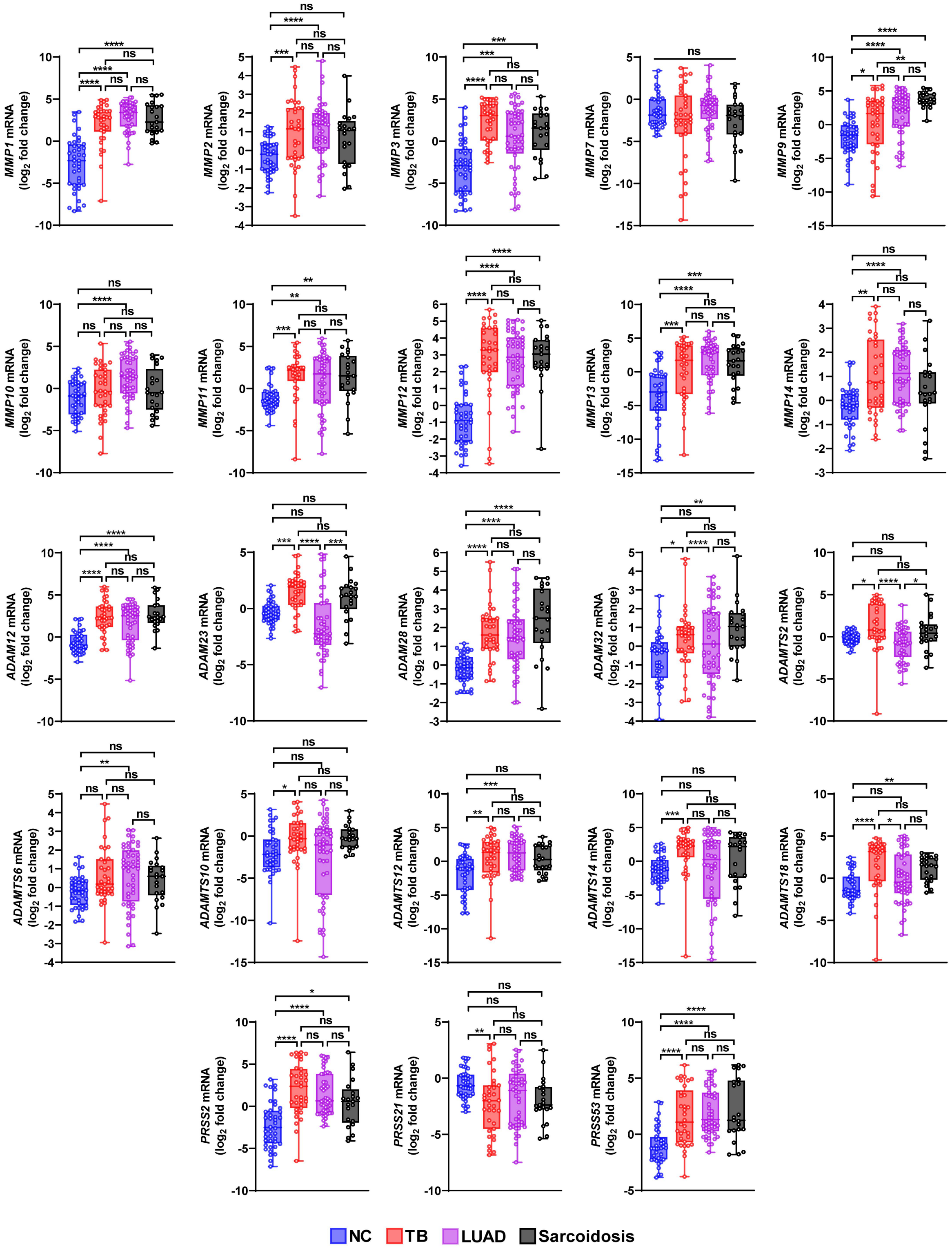

Supplementary Fig. 4 Expression of ECM protease-encoding genes in NC, TB, LUAD and sarcoidosis lung tissues. The mRNA levels of each indicated gene were determined by quantitative PCR analysis. Box-whisker plot indicates the interquartile range (box), the median value (line within the box) and the maximum and minimum value (whiskers). $P > 0.05$, not significant (ns); *$P < 0.05$; **$P < 0.01$; ***$P < 0.001$; ****$P < 0.0001$ (mean ± s.e.m. of n = 40, 35, 48 and 21 in NC, TB, LUAD and sarcoidosis groups, respectively, one-way ANOVA). Results are representatives from at least two independent experiments.

Supplementary Fig. 5

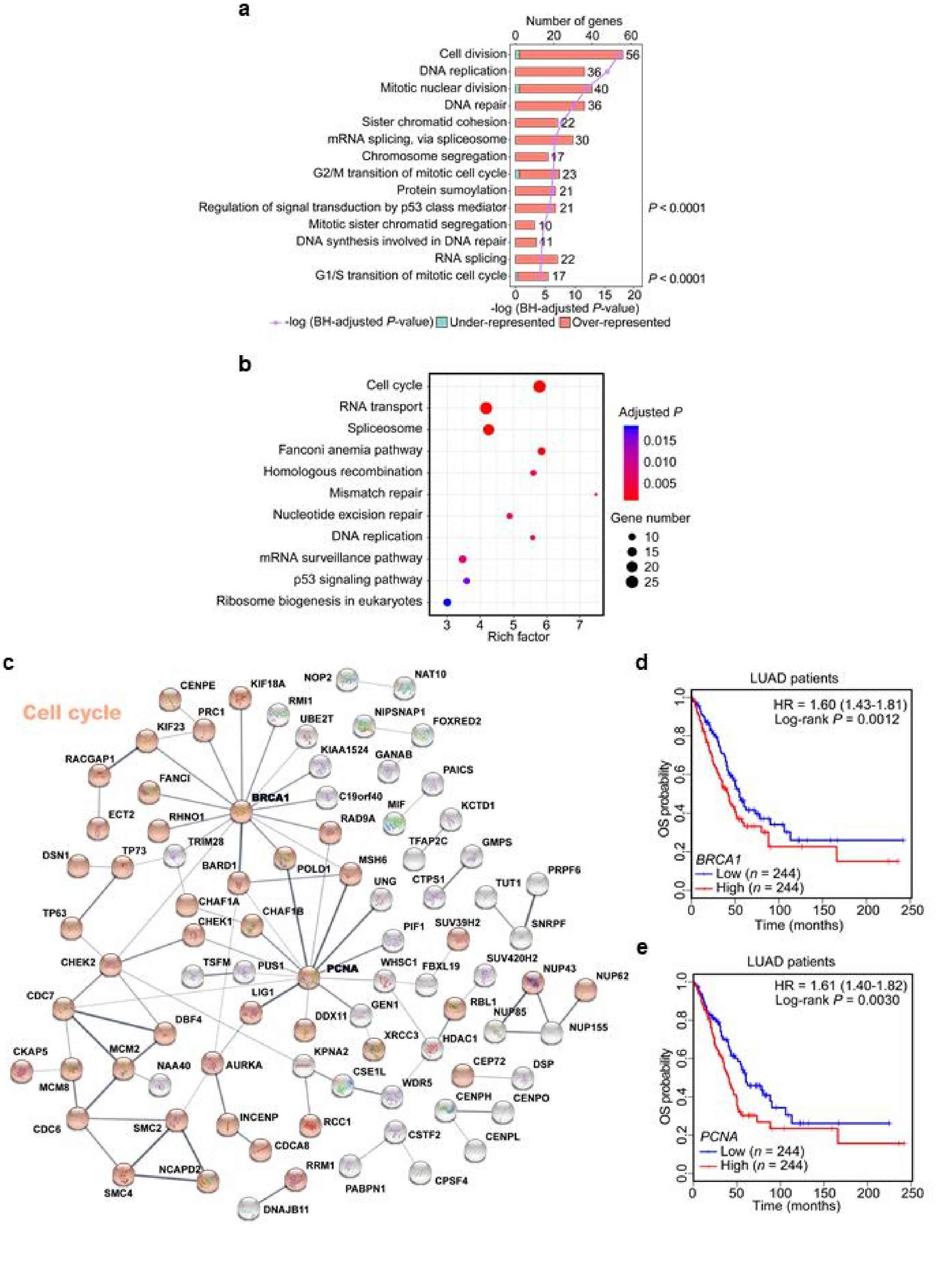

Supplementary Fig. 5 Enrichment of cell proliferation-related genes in LUAD lung tissues. **a** GO analysis showing the enrichment of cell proliferation-related genes in LUAD-correlated WGCNA gene module 21. The bars represent the abundance of under-represented (blue) and over-represent genes (red) in each biological process. The purple line represents BH-adjusted *P*-value for each biological process. **b** Kyoto Encyclopedia of Genes and Genomes (KEGG) pathway enrichment analysis of LUAD-correlated WGCNA gene module 21 showing the enrichment of cell proliferation-related gene pathways. **c** Interaction network analysis of DEGs of LUAD as compared to NC identified in LUAD-correlated WGCNA gene module 21. Genes associated with cell cycle according to GO enrichment analysis (Fisher's exact FDR = 1.22e-26) were colored in yellowish pink. Line thickness indicates the strength of data support. **d**, **e** The overall survival (OS) of LUAD patients from TCGA was compared between individuals with high or low levels of *BRCA1* mRNA (**d**) or *PCNA* mRNA (**e**) transcription. Hazard ratio (HR), 95% confidence interval and log-rank *P*-values are shown.

Supplementary Fig. 6

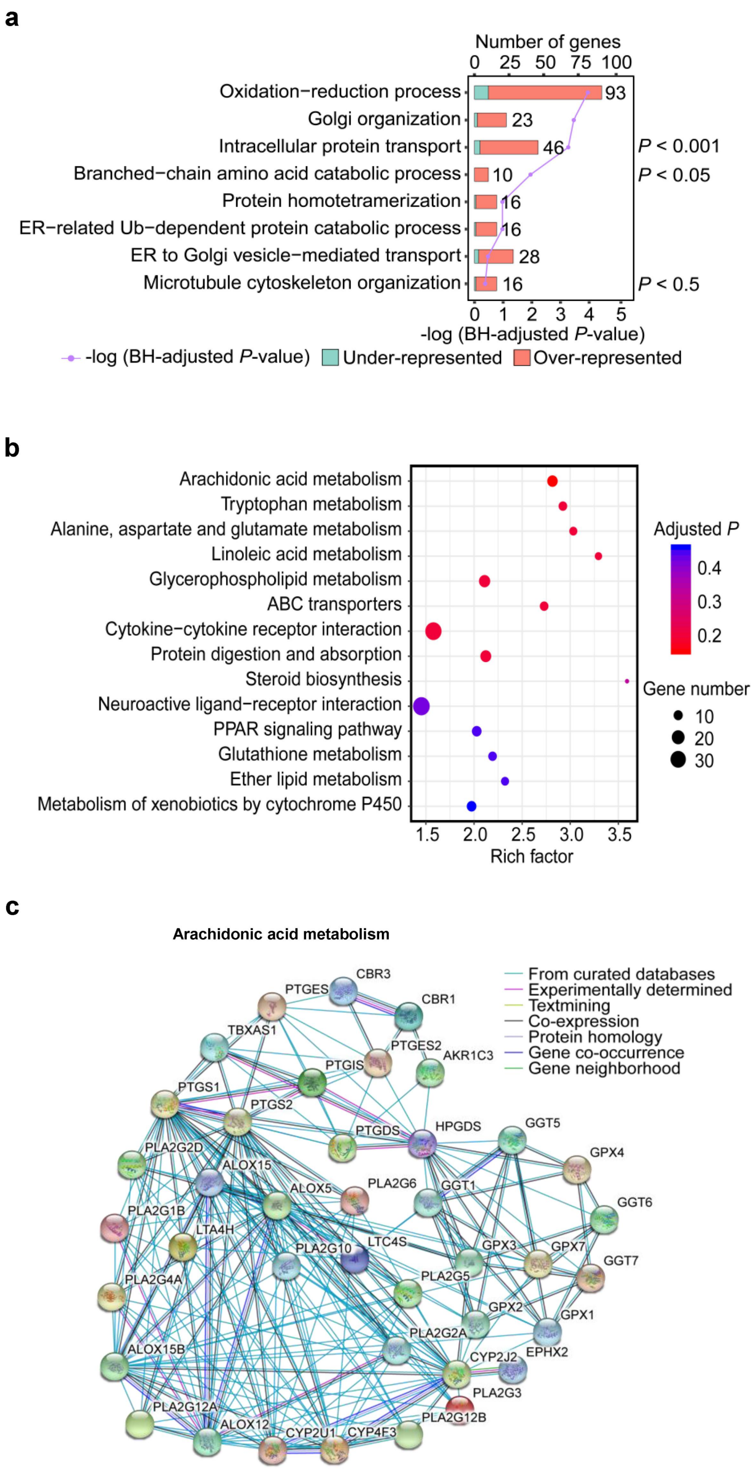

Supplementary Fig. 6 Enrichment of arachidonic acid metabolism-related genes in sarcoidosis lung tissues. a GO analysis showing the enrichment of oxidation-reduction process-related genes in sarcoidosis-correlated WGCNA gene module 26. The bars represent the abundance of under-represented (blue) and over-represent genes (red) in each biological process. The purple line represents BH-adjusted *P*-value for each biological process. **b** KEGG pathway enrichment analysis of DEGs of sarcoidosis as compared NC showing the enrichment of arachidonic acid metabolic pathway-related genes in sarcoidosis lung tissues. **c** Interaction network analysis of arachidonic acid metabolism-related genes in sarcoidosis -correlated WGCNA gene module 26.

Supplementary Fig. 7

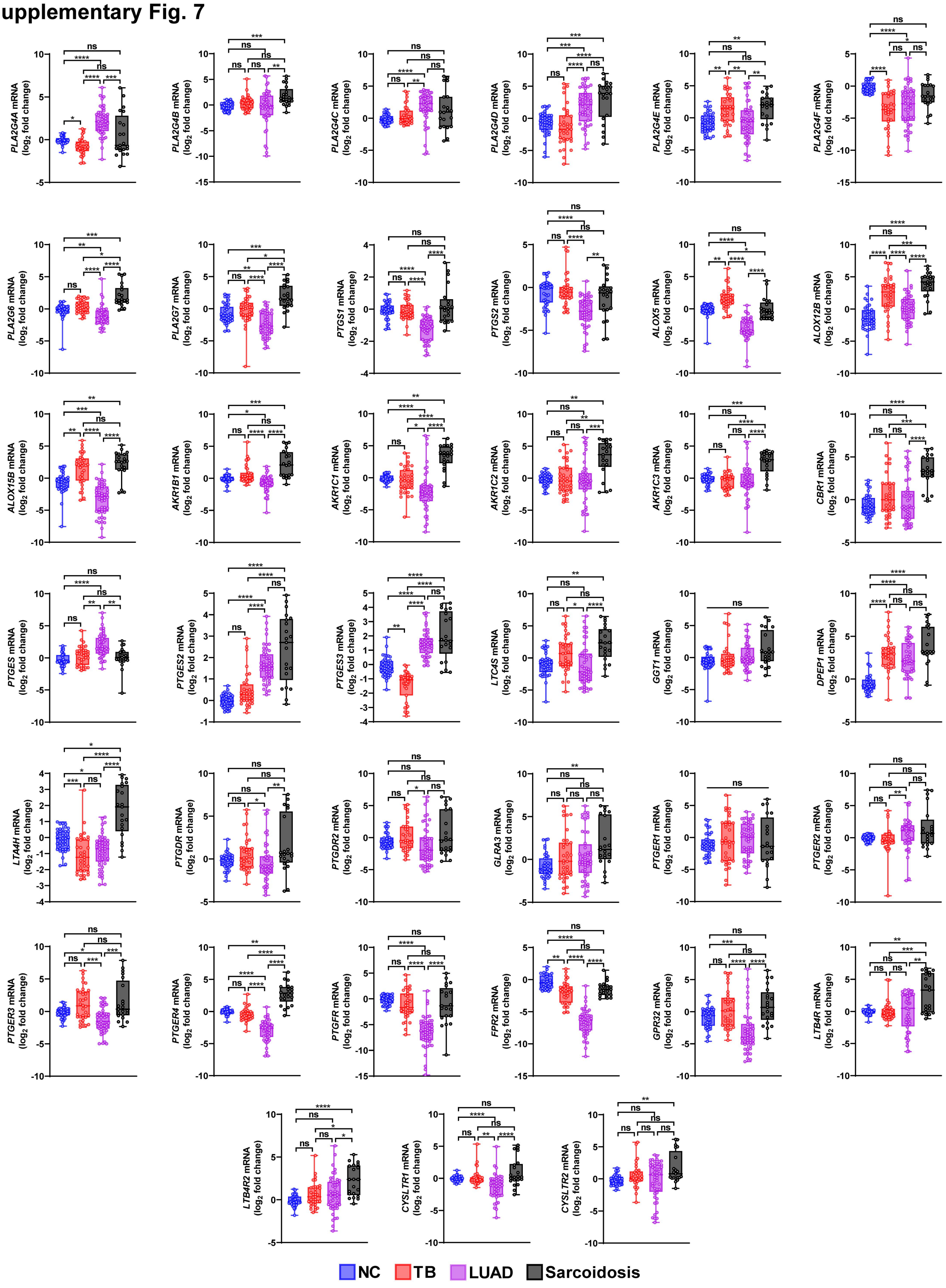

Supplementary Fig. 7 Expression of genes related to arachidonic acid metabolism in NC, TB, LUAD and sarcoidosis lung tissues. The mRNA levels of each indicated gene were determined by quantitative PCR analysis. Box-whisker plot indicates the interquartile range (box), the median value (line within the box) and the maximum and minimum value (whiskers). $P > 0.05$, not significant (ns); *$P < 0.05$; **$P < 0.01$; ***$P < 0.001$; ****$P < 0.0001$ (mean ± s.e.m. of n = 40, 35, 48 and 21 in NC, TB, LUAD and sarcoidosis groups, respectively, one-way ANOVA). Results are representatives from at least two independent experiments.

Supplementary Fig. 8

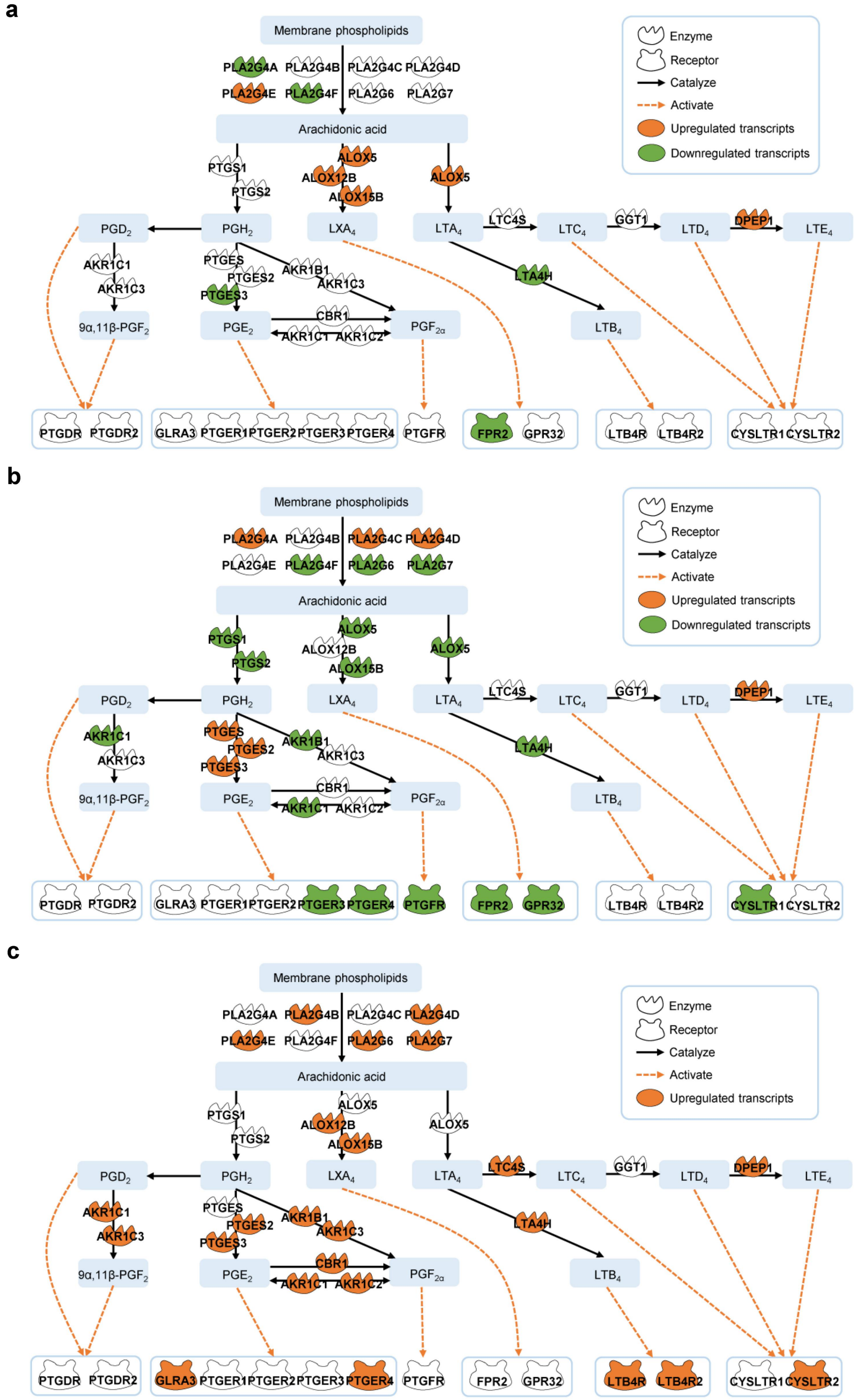

Supplementary Fig. 8 Characterization and expression of genes related to arachidonic acid metabolic pathway in TB, LUAD and sarcoidosis patients. The mRNA levels of indicated genes in TB (**a**), LUAD (**b**) and sarcoidosis (**c**) lung tissues were determined by quantitative PCR analysis. Colored genes are differentially expressed in TB, LUAD or sarcoidosis groups (n = 35, 48 and 21, respectively) as compared to NC group (n = 40) according to one-way ANOVA (Kruskal-Wallis test, $P < 0.05$).

Supplementary Fig. 9

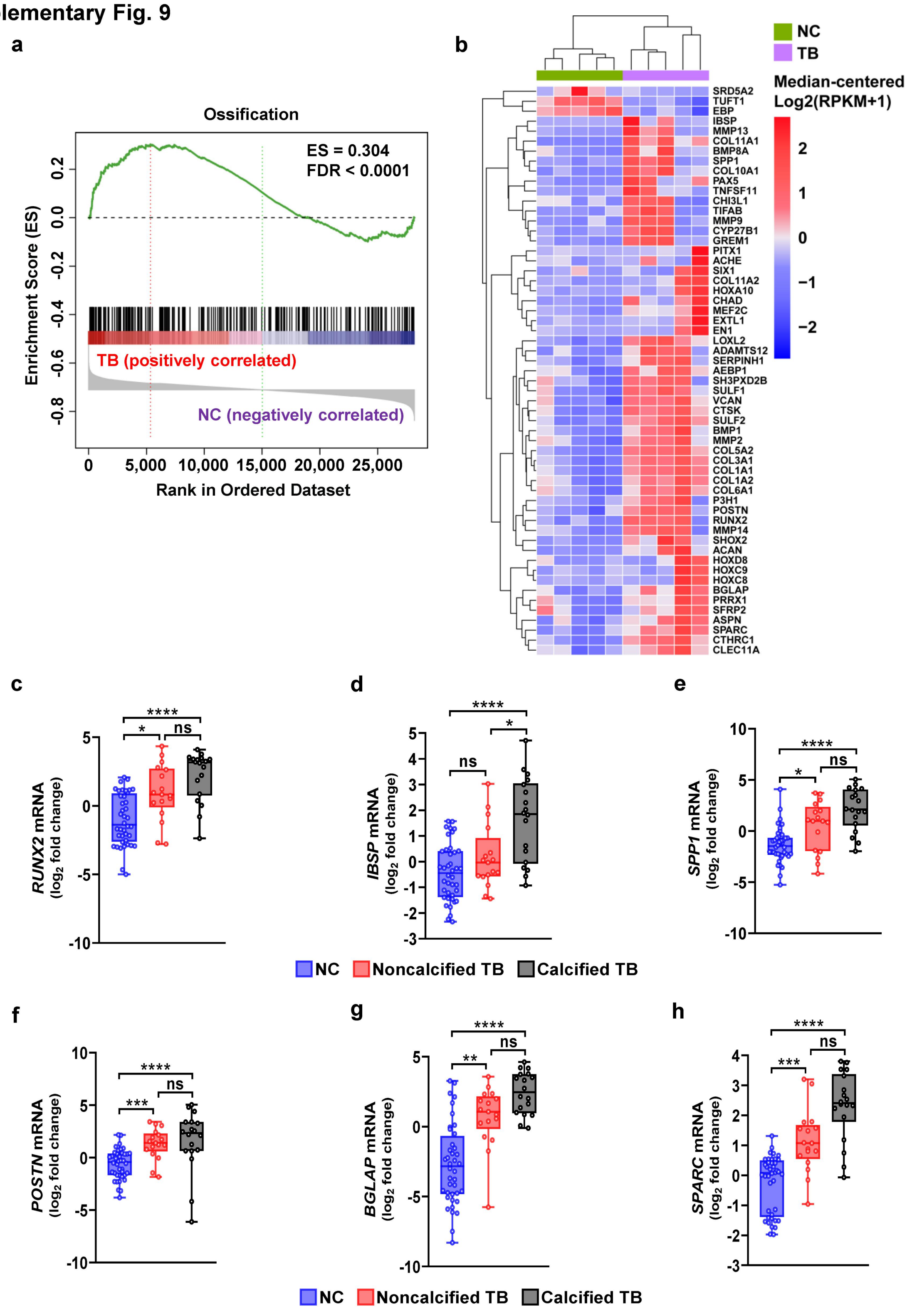

Supplementary Fig. 9 Increased expression of ossification-related genes in TB lung tissues. **a** GSEA of TB and NC lung-tissue transcriptional profiles showing the positive correlation of TB patients with ossification-related gene signatures. The bar-code plot indicates the positions of genes in the gene set, with red and blue bars representing upregulated and downregulated genes, respectively. **b** Heatmap depicting the expression of ossification-related genes in NC and TB lung tissues. These genes are involved in biological processes related to bone formation based on GO enrichment analysis (Fisher's exact FDR < 0.05) of DEGs of TB as compared to NC. **c–h** Quantitative PCR analysis of *RUNX2* (**c**), *IBSP* (**d**), *SPP1* (**e**), *POSTN* (**f**), *BGLAP* (**g**) and *SPARC* (**h**) mRNAs in NC, noncalcified TB or calcified TB lungs. Box-whisker plot indicates the interquartile range (box), the median value (line within the box) and the maximum and minimum value (whiskers). Comparisons were analyzed by one-way ANOVA (Kruskal-Wallis test). $P > 0.05$, not significant (ns); $^*P < 0.05$; $^{**}P < 0.01$; $^{***}P < 0.001$; $^{****}P < 0.0001$. Results are representatives from three independent experiments (n = 40, 17 and 18 in NC, noncalcified TB and calcified TB groups, respectively).

Supplementary Fig. 10

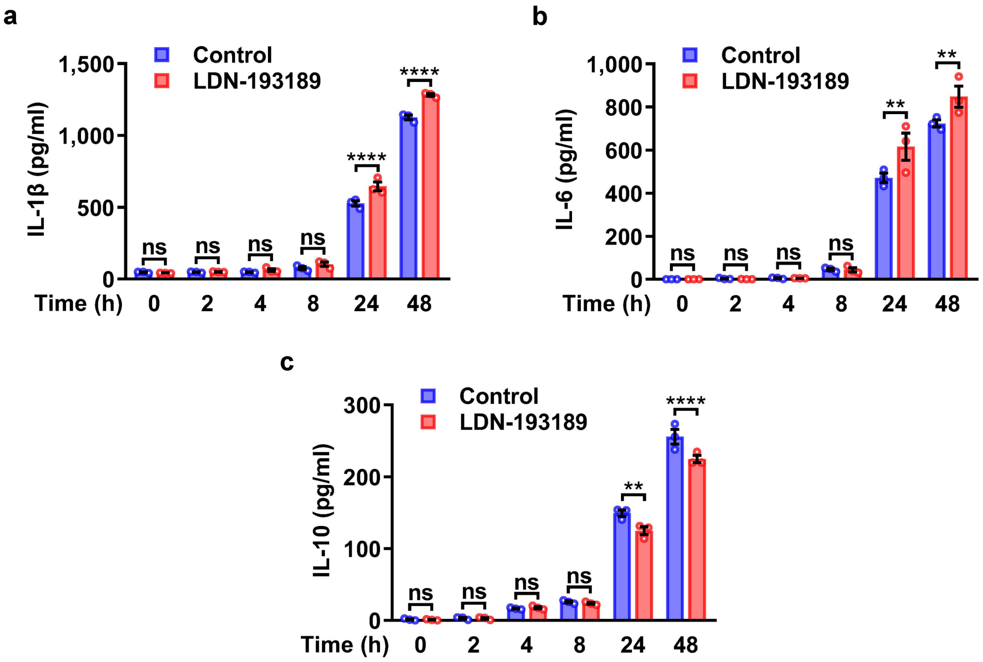

Supplementary Fig. 10 Treatment of LDN-193189 increases IL-1β and IL-6, and decreases IL-10 secreted by Mtb-infected macrophages. a–c Enzyme-linked immunosorbent assay of IL-1β (**a**), IL-6 (**b**) and IL-10 (**c**) in supernatants of U937 cells. Cells were treated with 500 nM LDN-193189 or control DMSO, and infected with Mtb H37Rv strain at MOI of 1 for 0–48 h. $P > 0.05$, not significant (ns); **$P < 0.01$; ****$P < 0.0001$ (n = 3, two-way ANOVA). Results are representatives from at least three independent experiments.

Supplementary Fig. 11

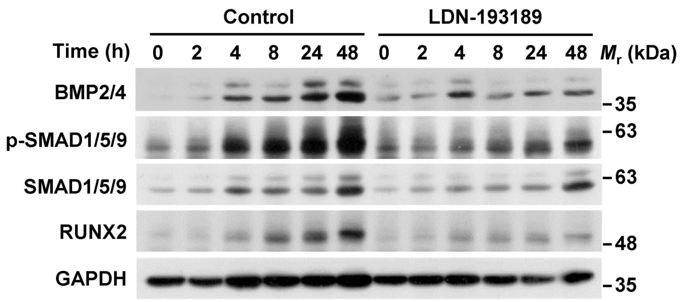

Supplementary Fig. 11 Mtb activates BMP/SMAD/RUNX2 signaling in macrophages. BMDMs were treated with 500 nM LDN-193189 or control DMSO, and infected with Mtb H37Rv strain at MOI of 1 for 0–48 h. Cells were then lysed for immunoblot analysis of BMP2/4, p-SMAD1/5/9, SMAD1/5/9, RUNX2 and GAPDH. Results are representatives from three independent experiments.

Supplementary Fig. 12

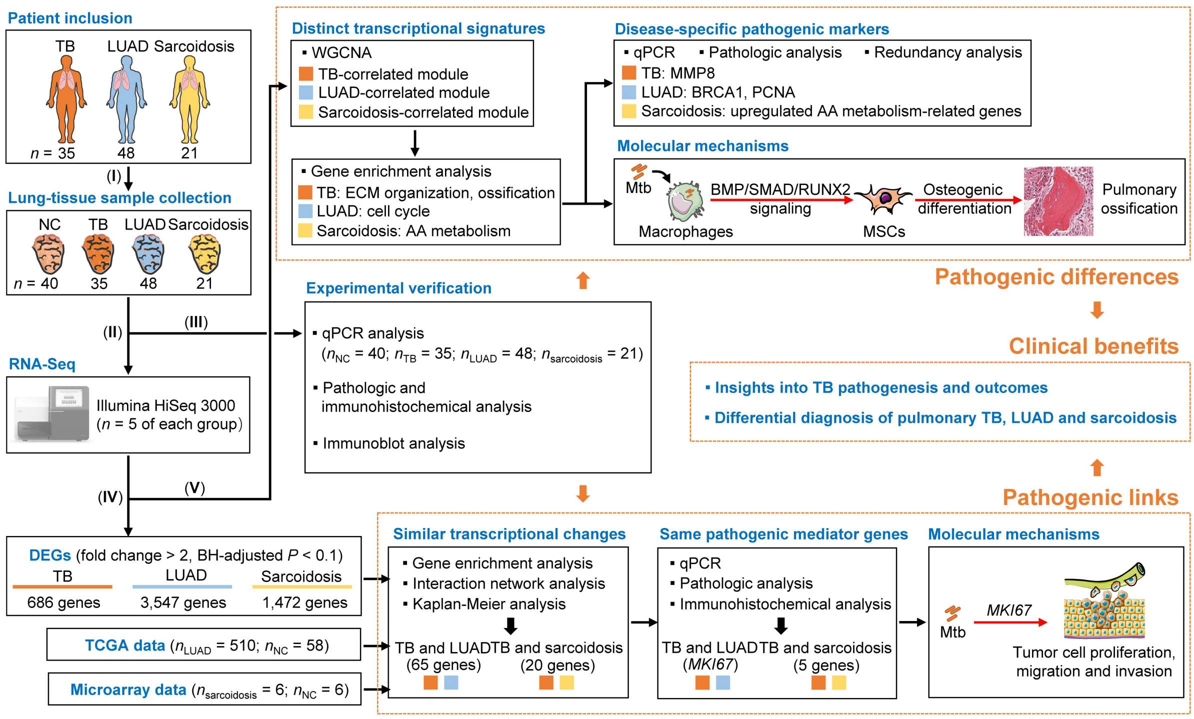

Supplementary Fig. 12 Schematic representation of the workflow for this study. A total of 104 patients including 35 TB patients, 48 LUAD patients and 21 sarcoidosis patients were included, and 144 lung samples including 40 NC, 35 TB, 48 LUAD and 21 sarcoidosis samples were collected (I). Lung samples were then used for transcriptional profiling by RNA-Seq (II) or subsequent experimental verification (III). On the one side, differentially expressed genes (DEGs) of TB, LUAD and sarcoidosis as compared to NC from RNA-Seq data, combined with lung-tissue transcriptional data of LUAD from TCGA and that of sarcoidosis from a published microarray study, were used for analysis of similar transcriptional changes among the diseases (IV). Same pathogenic mediator genes shared by TB and the other two diseases were identified to investigate their pathogenic links, among which *MKI67* was further confirmed to play an important role in Mtb-promoted tumor cell proliferation, migration and invasion. On the other side, RNA-Seq data were used for WGCNA followed by gene enrichment analysis to find distinct transcriptional signatures of each disease (V). Potential molecular markers were experimentally identified, and the mechanism by which Mtb causes pulmonary ossification in TB patients was further investigated. To conclude, these findings reveal new insights into TB pathogenesis, and provide potential molecular markers for differential diagnosis of pulmonary TB, LUAD and sarcoidosis.

Supplementary Fig. 13

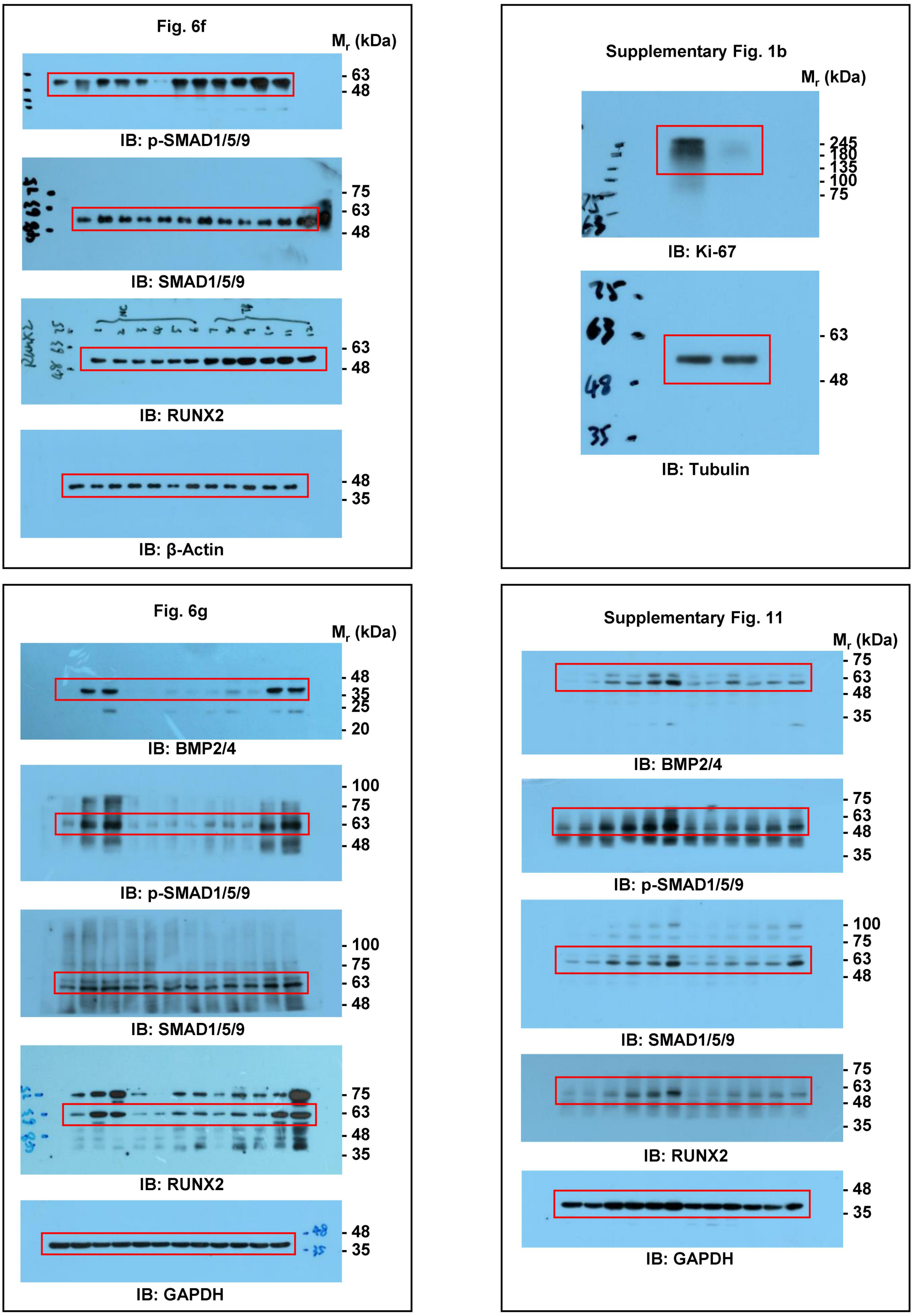

Supplementary Fig. 13 Original source data of the blot images. Cropped images indicated by red boxes were used for Fig. 6f, g and Supplementary Figs. 1b and 11.

Supplementary Table 1. Characteristics of patients for lung-tissue gene expression analysis by RNA-Seq.

Variable	NC (n = 5)	TB (n = 5)	LUAD (n = 5)	Sarcoidosis (n = 5)	*P*-value
**Age, years** (mean ± S.D.)	48.4 ± 17.2	45.8 ± 11.8	64.6 ± 9.8	34.6 ± 7.3	0.0329
**Gender** (male/female)	3/2	3/2	2/3	2/3	N.A.
**Smoking status** (former/never)	3/2	2/3	3/2	2/3	N.A.
**Confirmation of Mtb** (culture/Xpert/PCR)	N.A.	2/0/3	N.A.	N.A.	N.A.
**Pulmonary cavitation** (Yes/no)	0/5	2/3	0/5	0/5	N.A.
**Sign of calcification** (Yes/no)	2/3	3/2	0/5	3/2	N.A.
**Sign of fibrosis** (Yes/no)	1/4	1/4	0/5	2/3	N.A.
**Treatment before sampling** (Yes/no)	0/5	2/3	0/5	0/5	N.A.
**Tissue collection** (surgical resection/needle biopsy)	5/0	5/0	5/0	4/1	N.A.
**Sampling location**					N.A.
Left upper lobe	2	3	2	2	
Left lower lobe	0	0	0	0	
Right upper lobe	2	1	2	1	
Right middle lobe	0	0	0	0	
Right lower lobe	1	1	1	2	
**Tumor stage of LUAD**					N.A.
Stage I	N.A.	N.A.	1	N.A.	
Stage II	N.A.	N.A.	3	N.A.	
Stage III	N.A.	N.A.	1	N.A.	
Stage IV	N.A.	N.A.	0	N.A.	
**Roentgenographic stage of sarcoidosis**					N.A.
Stage I	N.A.	N.A.	N.A.	0	
Stage II	N.A.	N.A.	N.A.	0	
Stage III	N.A.	N.A.	N.A.	3	
Stage IV	N.A.	N.A.	N.A.	2	

NC, normal control; TB, tuberculosis; LUAD, lung adenocarcinoma; S.D., standard deviation; N.A., not applicable.

Supplementary Table 2. Characteristics of patients included in qPCR-based lung-tissue gene expression analysis.

**Variable**	**NC** (n = 40)	**TB** (n = 35)	**LUAD** (n = 48)	**Sarcoidosis** (n = 21)	***P*-value**
**Age, years** (mean ± S.D.)	55.4 ± 12.2	37.4 ± 12.0	60.4 ± 10.4	36.4 ± 13.4	< 0.0001
**Gender**					0.3175
Male (%)	17 (42.5)	18 (51.4)	19 (39.6)	13 (61.9)	
Female (%)	23 (57.5)	17 (48.6)	29 (60.4)	8 (38.1)	
**Smoking history**					0.7172
Former (%)	15 (37.5)	10 (28.6)	18 (37.5)	9 (42.9)	
Never (%)	25 (62.5)	25 (71.4)	30 (62.5)	12 (57.1)	
**Confirmation of Mtb**					N.A.
Culture (%)	N.A.	20 (57.1)	N.A.	N.A.	
Xpert (%)	N.A.	3 (8.6)	N.A.	N.A.	
PCR (%)	N.A.	12 (34.3)	N.A.	N.A.	
**Pulmonary cavitation** (%)	6 (15.0)	17 (48.6)	6 (12.5)	0 (0)	< 0.0001
**Sign of calcificatio**n (%)	8 (20.0)	18 (51.4)	3 (6.3)	5 (23.8)	< 0.0001
**Sign of fibrosis** (%)	9 (22.5)	17 (48.6)	9 (18.8)	9 (42.9)	0.0106
**Tissue collection**					< 0.0001
Surgical resection (%)	40 (100)	31 (88.6)	48 (100)	5 (23.8)	
Needle biopsy (%)	0 (0)	4 (11.4)	0 (0)	16 (76.2)	
**Sampling location**					0.8235
Left upper lobe (%)	12 (30.0)	11 (31.4)	13 (27.1)	4 (19.0)	
Left lower lobe (%)	3 (7.5)	6 (17.1)	8 (16.7)	4 (19.0)	
Right upper lobe (%)	13 (32.5)	7 (20)	14 (29.2)	5 (23.8)	
Right middle lobe (%)	5 (12.5)	2 (5.7)	4 (8.3)	1 (4.8)	
Right lower lobe (%)	7 (17.5)	9 (25.7)	9 (18.8)	7 (33.3)	
**Tumor stage of LUAD**					N.A.
Stage I (%)	N.A.	N.A.	25 (52.1)	N.A.	
Stage II (%)	N.A.	N.A.	11 (20.8)	N.A.	
Stage III (%)	N.A.	N.A.	10 (20.8)	N.A.	
Stage IV (%)	N.A.	N.A.	2 (6.3)	N.A.	
**Roentgenographic stage of sarcoidosis**					N.A.
Stage I (%)	N.A.	N.A.	N.A.	0 (0)	
Stage II (%)	N.A.	N.A.	N.A.	0 (0)	
Stage III (%)	N.A.	N.A.	N.A.	12 (57.1)	
Stage IV (%)	N.A.	N.A.	N.A.	9 (42.9)	

NC, normal control; TB, tuberculosis; LUAD, lung adenocarcinoma; S.D., standard deviation; N.A., not applicable.
