# Supplementary material for: Lung gene expression signatures suggest pathogenic links and molecular markers for pulmonary tuberculosis, adenocarcinoma and sarcoidosis
Source: Commun Biol. 2020 Oct 23;3:604. doi: 10.1038/s42003-020-01318-0 (PMC7584606; doi:10.1038/s42003-020-01318-0)
Supplement: Supplementary file 2 — Description of Additional Supplementary Files [file 42003_2020_1318_MOESM2_ESM.pdf]

Description of Additional Supplementary Files

Supplementary Data 1. Demographic and clinical characteristics of patients included in this study.

Supplementary Data 2. A total of 686 differentially expressed genes of tuberculosis group as compared to the normal control group.

Supplementary Data 3. A total of 3547 differentially expressed genes of lung adenocarcinoma group as compared to the normal control group.

Supplementary Data 4. A total of 1472 differentially expressed genes of sarcoidosis group as compared to the normal control group.

Supplementary Data 5. A total of 65 genes significantly correlated with lung adenocarcinoma patient survival and similarly expressed in tuberculosis and adenocarcinoma lungs.

Supplementary Data 6. A total of 20 genes similarly expressed in tuberculosis and sarcoidosis lung tissues.

Supplementary Data 7. Primer sequences used for quantitative PCR in this study.

Supplementary Data 8. Gene Ontology term enrichment for modular genes in weighted correlation network analysis.

Supplementary Data 9. Source data underlying the graphs in this study.
