# Supplementary material for: Lung gene expression signatures suggest pathogenic links and molecular markers for pulmonary tuberculosis, adenocarcinoma and sarcoidosis
Source: Commun Biol. 2020 Oct 23;3:604. doi: 10.1038/s42003-020-01318-0 (PMC7584606; doi:10.1038/s42003-020-01318-0)
Supplement: Supplementary file 12 — Reporting Summary [file 42003_2020_1318_MOESM12_ESM.pdf]

nature research

Corresponding author(s): Cui Hua Liu

Last updated by author(s): Sep 18, 2020

Reporting Summary

Nature Research wishes to improve the reproducibility of the work that we publish. This form provides structure for consistency and transparency in reporting. For further information on Nature Research policies, see Authors & Referees and the Editorial Policy Checklist.

Statistics

For all statistical analyses, confirm that the following items are present in the figure legend, table legend, main text, or Methods section.

n/a	Confirmed	
☐	☒	The exact sample size (*n*) for each experimental group/condition, given as a discrete number and unit of measurement
☐	☒	A statement on whether measurements were taken from distinct samples or whether the same sample was measured repeatedly
☐	☒	The statistical test(s) used AND whether they are one- or two-sided<br>*Only common tests should be described solely by name; describe more complex techniques in the Methods section.*
☒	☐	A description of all covariates tested
☐	☒	A description of any assumptions or corrections, such as tests of normality and adjustment for multiple comparisons
☐	☒	A full description of the statistical parameters including central tendency (e.g. means) or other basic estimates (e.g. regression coefficient) AND variation (e.g. standard deviation) or associated estimates of uncertainty (e.g. confidence intervals)
☐	☒	For null hypothesis testing, the test statistic (e.g. *F*, *t*, *r*) with confidence intervals, effect sizes, degrees of freedom and *P* value noted<br>*Give P values as exact values whenever suitable.*
☒	☐	For Bayesian analysis, information on the choice of priors and Markov chain Monte Carlo settings
☒	☐	For hierarchical and complex designs, identification of the appropriate level for tests and full reporting of outcomes
☒	☐	Estimates of effect sizes (e.g. Cohen's *d*, Pearson's *r*), indicating how they were calculated

Our web collection on statistics for biologists contains articles on many of the points above.

Software and code

Policy information about availability of computer code

Data collection	FASTQC 0.11.3 and Trimmomatic 0.39 were used to trim adaptors and low quality bases; STAR 2.4.2a was used for alignment of trimmed reads to the ensembl 79 (GRCh38.p2) reference genome; FeatureCounts 1.6.2 was used to generate expression matrices for downstream analysis.
Data analysis	GraphPad Prism 8.0 and R 3.4.4 were used for statistics; DEseq2 1.18.1 and limma 3.34.9 were used to determine differential expression; Pheatmap 1.0.12 was used to perform cluster analysis; survival 3.1.8 was used for overall survival analysis; ClusterProfiler 3.6.0 was used to perform enrichment analysis; GSEA source code was obtained from the original publication (http://software.broadinstitute.org/gsea/msigdb/index.jsp); Protein–protein interaction network was constructed by STRING database; WGCNA 1.68 and Cytoscape 3.6.0 were used to construct co-expression network; Vegan 2.5.6 was used for redundancy analysis; RandomForest 4.6.14 was used to calculate Gini score; IBM SPSS Statistics 22.0 was used to perform ROC analysis.

For manuscripts utilizing custom algorithms or software that are central to the research but not yet described in published literature, software must be made available to editors/reviewers. We strongly encourage code deposition in a community repository (e.g. GitHub). See the Nature Research guidelines for submitting code & software for further information.

Data

Policy information about availability of data

All manuscripts must include a data availability statement. This statement should provide the following information, where applicable:

- Accession codes, unique identifiers, or web links for publicly available datasets
- A list of figures that have associated raw data
- A description of any restrictions on data availability

The source data underlying Figs. 2b, c, e, g, h–l, 3b, d, f, g, i, 5a, b, d, e, f, g, i, j, 6d, e, h–j and m–o, and Supplementary Figs. 1a, c, d, f, h, j, 4, 7, 9c–h and 10 were provided in Supplementary Data 9. The original data of unprocessed blot images were provided in Supplementary Fig. 13. The RNA sequencing data from this study have been deposited to the NCBI Sequence Read Archive and are accessible through GEO Series accession number GSE148036 (https://www.ncbi.nlm.nih.gov/geo/

query/acc.cgi?acc=GSE148036). All other data that support the findings of this study are available from the corresponding author upon reasonable request.

Field-specific reporting

Please select the one below that is the best fit for your research. If you are not sure, read the appropriate sections before making your selection.

☒ Life sciences ☐ Behavioural & social sciences ☐ Ecological, evolutionary & environmental sciences

For a reference copy of the document with all sections, see nature.com/documents/nr-reporting-summary-flat.pdf

Life sciences study design

All studies must disclose on these points even when the disclosure is negative.

Sample size	For RNA-Seq library preparation and sequencing, 5 independent lung samples were sufficient to represent the signature of transcriptional profiles of each group and for subsequent exploratory analysis. Disease signature genes from RNA-Seq data were confirmed and reproduced in more than 20 independent lung samples for each group by quantitative PCR analysis, and/or in at least 8 independent lung samples for each group by immunohistochemical analysis. For all experiments of molecular biology and biochemistry in this study, three independent experiments and each with 3-6 independent biological replicates were performed, which could be sufficient to determine the differences between the tested groups.
Data exclusions	None.
Replication	Experiments were repeated at least three independent times. No experiment was found to be irreproducible.
Randomization	Individuals were allocated into experimental groups by clinical criteria as described in the manuscript. Randomization was not relevant to our study as we were not conducting an interventional study.
Blinding	Blinding was not relevant to our study, as there were no control and treatment arms involved.

Reporting for specific materials, systems and methods

We require information from authors about some types of materials, experimental systems and methods used in many studies. Here, indicate whether each material, system or method listed is relevant to your study. If you are not sure if a list item applies to your research, read the appropriate section before selecting a response.

Materials & experimental systems

n/a	Involved in the study	
☐	☒	Antibodies
☐	☒	Eukaryotic cell lines
☒	☐	Palaeontology
☐	☒	Animals and other organisms
☐	☒	Human research participants
☒	☐	Clinical data

Methods

n/a	Involved in the study	
☒	☐	ChIP-seq
☒	☐	Flow cytometry
☒	☐	MRI-based neuroimaging

Antibodies

Antibodies used	Rabbit anti-PtpA antibody was produced and purified as described in a previously published work from us (J. Wang et al., 2015). The following commercially available antibodies were used in this study: anti-collagen I (#NB600-408, Novus Biologicals, 1:400 for immunohistochemical staining), anti-collagen III (#ab2345, Abcam, 1:200 for immunohistochemical staining), anti-Ki-67 (#NB500-170, Novus Biologicals, 1:100 for immunohistochemical staining and 1:2000 for immunoblot analysis), anti-Cathepsin K (#sc-48353, Santa Cruz, 1:200 for immunohistochemical staining), anti-MMP8 (#sc-514803, Santa Cruz, 1:100 for immunohistochemical staining), anti-BRCA1 (#ab16780, Abcam, 1:100 for immunohistochemical staining), anti-PCNA (#sc-25280, Santa Cruz, 1:100 for immunohistochemical staining), anti-p-SMAD1/5/9 (#13820, Cell Signaling Technology, 1:100 for immunohistochemical staining and 1:1000 for immunoblot analysis), anti-SMAD1/5/9 (#ab66737, Abcam, 1:1000 for immunoblot analysis), anti-RUNX2 (#sc-101145, Santa Cruz, 1:100 for immunohistochemical staining and 1:2000 for immunoblot analysis), anti-BMP2/4 (#sc-137087, Santa Cruz, 1:1000 for immunoblot analysis), anti-GAPDH (#sc-25778, Santa Cruz, 1:4000 for immunoblot analysis), anti-β-actin (#A2228, Sigma-Aldrich, 1:4000 for immunoblot analysis), anti-Tubulin (#T5168; Sigma-Aldrich), anti-CD29 (#sc-9970, Santa Cruz, 1:100 for immunohistochemical staining) and anti-CD68 (#sc-17832, Santa Cruz, 1:100 for immunohistochemical staining).
Validation	Validation statements for antibodies used for immunohistochemical staining (collagen I, collagen III, Ki-67, BRCA1, PCNA, Cathepsin K, MMP8, BRCA1, PCNA, p-SMAD1/5/9, RUNX2, CD29 and CD68) and immunoblot analysis (Ki-67, p-SMAD1/5/9, SMAD1/5/9, RUNX2, BMP2/4, GAPDH and β-actin) can be found on their corresponding manufacturer websites. Rabbit anti-PtpA antibody has been confirmed by using the sample of whole bacterial cell lysate of Mtb for immunoblot detection of a specific

band at the predicted size with a negative control of the preimmune serum, and has also been validated for the use of immunofluorescent and immunohistochemical analysis of Mtb-infected mammalian cells in our previously published works (J. Wang et al., 2015 and 2017).

Eukaryotic cell lines

Policy information about cell lines

Cell line source(s)	A549 cells (ATCC CCL-185) and U937 cells (ATCC CRL-1593.2) were obtained from the American type culture collection (ATCC).
Authentication	No further authentication was made.
Mycoplasma contamination	All cell lines were tested negative for mycoplasma contamination.
Commonly misidentified lines (See ICLAC register)	No misidentified cell lines were used in this study.

Animals and other organisms

Policy information about studies involving animals; ARRIVE guidelines recommended for reporting animal research

Laboratory animals	Bone marrow-derived macrophages and mesenchymal stem cells were collected from 7–8 weeks old mice on C57BL/6 genetic background.
Wild animals	This study did not involve wild animals.
Field-collected samples	This study did not involve field-collected samples.
Ethics oversight	All mice were housed in a specific pathogen-free (SPF) facility on the basis of standard humane animal husbandry protocols, which were approved by the animal care and use committee of the Institute of Microbiology (Chinese Academy of Sciences). All animal studies were approved by the Biomedical Research Ethics Committee of Institute of Microbiology (Chinese Academy of Sciences).

Note that full information on the approval of the study protocol must also be provided in the manuscript.

Human research participants

Policy information about studies involving human research participants

Population characteristics	The demographic and clinical characteristics of all included patients were list in Supplementary Data 1. Statistical analysis of patient characteristics were list in Supplementary Table 1 (for RNA-Seq sequencing) and Supplementary Table 2 (for quantitative PCR analysis).
Recruitment	Patients were recruited between August 2017 and April 2019, from Beijing Chest Hospital, beijing, China. Finally, a total of 35 pulmonary tuberculosis patients, 48 lung adenocarcinoma patients and 21 pulmonary sarcoidosis patients from mainland China who received lung resection surgery or diagnostic biopsy were included into corresponding experimental groups by clinical criteria as described in the "Methods" section.
Ethics oversight	Ethical permission for this study was obtained from the ethics committee of Beijing Chest Hospital and Capital Medical University, Beijing, China.

Note that full information on the approval of the study protocol must also be provided in the manuscript.
